# Grain-Boundary Interaction between Inconel 625 and WC during Laser Metal Deposition

**DOI:** 10.3390/ma11101797

**Published:** 2018-09-21

**Authors:** Jan Huebner, Dariusz Kata, Paweł Rutkowski, Paweł Petrzak, Jan Kusiński

**Affiliations:** 1 Faculty of Materials Science and Ceramics, Department of Ceramics and Refractories, AGH University of Science and Technology, al. Mickiewicza 30, 30-059 Krakow, Poland; kata@agh.edu.pl (D.K.); pawelr@agh.edu.pl (P.R.); 2 Faculty of Metals Engineering and Industrial Computer Science, Department of Surface Engineering and Materials Characterisation, AGH University of Science and Technology, al. Mickiewicza 30, 30-059 Krakow, Poland; ppetrzak@agh.edu.pl (P.P.); kusinski@agh.edu.pl (J.K.)

**Keywords:** metal matrix composites, laser metal deposition, Inconel 625, additive manufacturing, laser processing

## Abstract

In this study, the laser metal deposition (LMD) of the Inconel 625–tungsten carbide (WC) metal matrix composite was investigated. The composite coating was deposited on Inconel 625 substrate by powder method. A powder mixture containing 10 wt% of WC (5 µm) was prepared by wet mixing with dextrin binder. Coating samples obtained by low-power LMD were pore- and crack-free. Ceramic reinforcement was distributed homogenously in the whole volume of the material. Topologically close-packed (TCP) phases were formed at grain boundaries between WC and Inconel 625 matrix as a result of partial dissolution of WC in a nickel-based alloy. Line analysis of the elements revealed very small interference of the coating in the substrate material when compared to conventional coating methods. The average Vickers hardness of the coating was about 25% higher than the hardness of pure Inconel 625 reference samples.

## 1. Introduction

A constantly increasing need for improvement in the field of energy harvesting has resulted in much research focused on developing innovation. Materials that can be used in high temperatures are promising because of possible applications in powerplants, and the aerospace and chemical industry [1,2,3]. The easiest way to improve the effectiveness of gas turbines used in engines is to elevate their work temperature. Today, widely used metallic materials allow for the production of turbine blades that are able to operate in temperatures in the range of 650–1200 °C. Additionally, these parts are constantly exposed to chemical and mechanical factors [4,5]. During their work, an aggressive environment causes microcavities in the material that may cause the complete destruction of working parts. Turbine blades are made of heat-resistant steel or nickel/cobalt-based superalloys. Because of the harsh environment and relatively short lifespan of the blades, any opportunity to regenerate damaged element is very attractive [6,7,8,9].

Nickel and cobalt superalloys are efficiently used in the production of parts for high-temperature applications. Their properties, excellent weldability, high plasticity, and corrosion/wear-resistance in high temperatures [10,11,12,13,14,15,16], are suitable for use in high-temperature environments. In order to improve the quality and lifespan of the used materials, metal matrix composites (MMC) with carbide reinforcement were proposed. In MMCs, the desirable properties of metals and ceramics are fused to obtain improved material. The combination of coating and substrate material can be designed to enhance specific properties: corrosion, oxidation, erosion, and high wear resistance [4,5,17,18].

Production of whole parts from materials that are characterized by good wear resistance is expensive due to the high cost of alloying elements such as Ni, Co, Mo, V, and W. A possible solution is surface modification by the deposition of protective coating. Deposition technology has had a huge impact on the fretting resistance of coatings. Surface layers produced from the same material but by different techniques vary in physical and performance properties. Industrial production of metal–ceramic composites can be done by laser metal deposition [18,19], thermal spraying [19,20,21] and plasma arc welding.

Some research investigated the laser processing of Inconel–carbide systems. It is reported that the mechanical properties of LMD-deposited materials are improved when compared to pure alloys [22,23,24,25,26]. Thanks to rapid solidification, the microstructure of the material is much finer when compared to conventional methods, such as Tungsten Inert Gas. Pure Inconel alloys are strengthened by the γ’ and γ” phases, which precipitate from austenitic γ-Ni during heat treatment. The δ phase (Ni_3_Nb) can also precipitate from alloys with a high Nb content. Phase δ helps refine the grain size and impeding dislocation in the structure. Introduction of tungsten carbide (WC) grains with a diameter over 10 µm into the system [27,28] shows higher wear resistance and hardness of the material. It was reported that big carbide particles could not be distributed homogenously throughout the whole volume of the sample. This results in non uniform distribution of hardness. Metal–carbide systems are commonly deposited using two separate powder feeders with regulated feed ratios that could not provide the same carbide content in the whole volume [27,28]. Introduction of a small amount of TiC nanoparticles into Inconel [29,30,31] resulted in a modified microstructure of the material. Hardness was improved due to grain-size refinement. Such a microstructure improves tensile properties of the composite.

In this research, we propose the use of nickel-based MMC protective coatings with ceramic reinforcement with a diameter about 5 µm. Rapid prototyping, laser metal deposition (also called laser cladding), was used as a deposition method [32,33]. Due to precise heating of a small part of material surface, it is possible to avoid interference in the substrate microstructure and chemical composition. High-energy density of a laser beam enables fast heating followed by rapid cooling and solidification coating. As a result, the obtained microstructure is characterised by fine grains and is resistant to erosion [27,28,34]. In this study, the grain-boundary Inconel 625–WC interaction was investigated during laser metal deposition. The phenomenon that occurs on the interface between these two different types of materials is crucial for understanding the nature of the LMD process. Moreover, obtained results can lead to easier implementation of this method to other composite systems. In this work, interaction between WC grains (5 µm) and a nickel-based superalloy during high-temperature LMD was investigated. The particle size of 5 µm was chosen in order to achieve uniform carbide distribution in the whole volume of the material. Additionally, this grain size allowed to avoid dissolution of a significant amount of WC in the metal matrix [35,36,37,38]. The powder mixture of Inconel 625 and WC was used to enhance homogenous distribution of carbide in the material.

## 2. Materials and Methods

**Figure 1:**
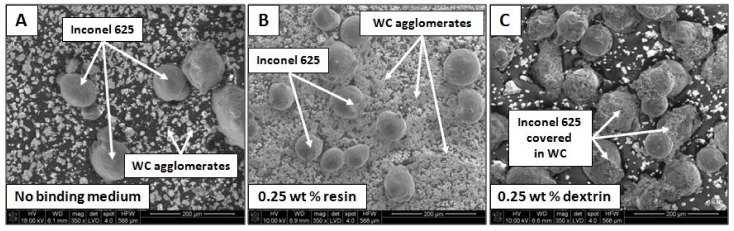
Scanning electron microscopy (SEM) images of powder mixture after each stage of homogenization: (**A**) no binder, (**B**) 0.25 wt% of resin binder, (**C**) 0.25 wt% of dextrin binder.

The experiments were performed by use of a powder mixture instead of two separate powders. It was obtained by homogenization of commercially available WC and Inconel 625. Inconel 625 powder, with an average particle diameter of 104 µm, and WC powder, with an average particle diameter of 5 µm, were mixed in a 9:1 mass proportion that resulted in 10 wt% of WC in mixture. Powders were initially homogenized for 90 min in a ball mill using WC balls in a weight ratio of 1:1 (grinding media:powder). In order to improve adhesion between Inconel 625 and WC particles, 0.25 wt% of resin was added and homogenization was repeated. Further addition of 0.25 wt% of dextrin was needed to achieve desired adhesion between powders after process. The morphology of the powder mixture at each step is shown in Figure 1.

**Figure 2:**
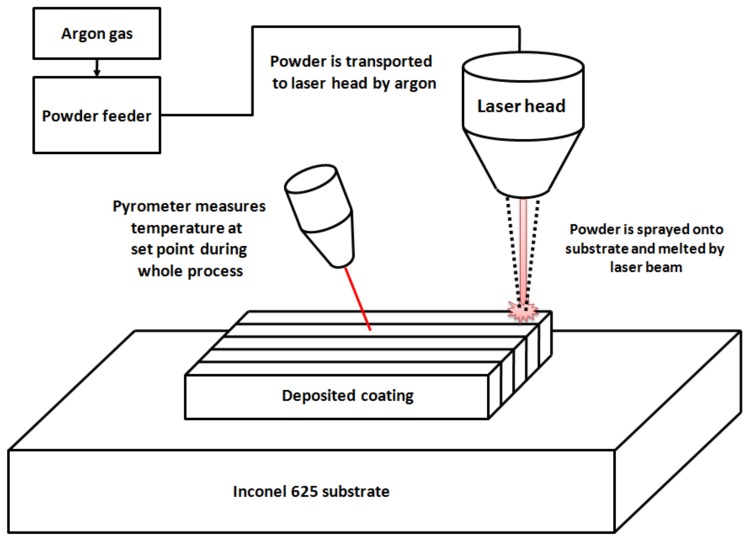
Schematic representation of the laser metal deposition process.

JK Laser Company model JK2000FL equipped with ytterbium-doped fiber was used to perform the laser metal deposition of the composite. Metal matrix composite coating was obtained by powder-mixture deposition on the Inconel 625 substrate. It was chosen to prevent additional impurities in the samples. To obtain a coating of 10 × 10 mm and of about 1 mm thickness, 10 subsequent tracks with a width of about 1 mm were deposited in 6 sublayers. The powder mixture was transported in protective atmosphere of argon from the powder feeder to the laser head and then sprayed onto the substrate. The powder particles melted due to exposure to the high-energy laser beam. The formation of a melt pool containing both the powder mixture and substrate material was observed. To avoid the decomposition of the ceramic reinforcement (WC melting point at 2870 °C), low power of the laser (320 W) was used. This enabled melting the Inconel 625 matrix (melting point at1340 °C). A radiation pyrometer monitored temperature changes during the LMD process at one point on the surface. Solidification of the material proceeded as the laser head moved with a set scanning speed. Samples were prepared by LMD according to the parameters presented in Table 1. Additionally, pure Inconel 625 reference samples were prepared. The schematic representation of process is shown in Figure 2.

Samples were cut in parallel and perpendicularly to the deposited tracks and then ground and polished. In order to observe the microstructure, samples were electrochemically etched in a 10% CrO_3_ water solution. 

Scanning electron microscopy (SEM) observations and energy-dispersive X-ray analysis were performed on a HITACHI S-3500N microscope equipped with an EDS NORAN 986B-1SPS analyzer, and an FEI Inspect S50 microscope with an EDAX EDS analyzer. To check the phase composition of the samples, X-ray diffraction analysis was performed using a PANalitycal X-ray Diffractor (XRD) equipped with Cu tube and X-pert HighScore software. The angle of the XRD ranged from 5° to 90° with a 0.008° measurement step. Transmission electron microscopy (TEM) observations were performed using a 200 kV JEAOL JEM-2010ARP microscope. Additionally, TEM-EDS analysis was done to check the elemental composition of the precipitates. Hardness was measured with a Future-Tech FM-700 hardness tester with a Vickers indenter under a load of 200 g for 15 s.

## 3. Results

**Figure 3:**
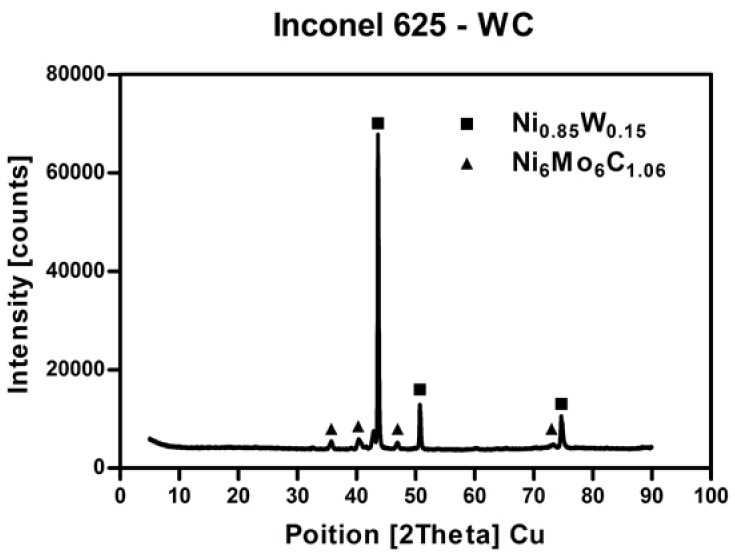
X-ray diffraction analysis of obtained Inconel 625–WC composite.

X-ray diffraction analysis is presented in Figure 3. The performed XRD analysis revealed the presence of two major phases: Ni_0.85_W_0.15_ and Ni_6_Mo_6_C_1.06_. This indicates that WC grains could be dissolved in a Ni matrix. The same carbide behavior was reported in different types of similar austenitic structures [30,31]. During that process, W diffused inside the γ-Ni matrix, while carbon remained in the intergranular region. It enabled the formation of a small amount of secondary carbides. The level of detection in the XRD technique varied from about 5%; thus, there is a possibility that other undetected phases are present in the material.

**Figure 4:**
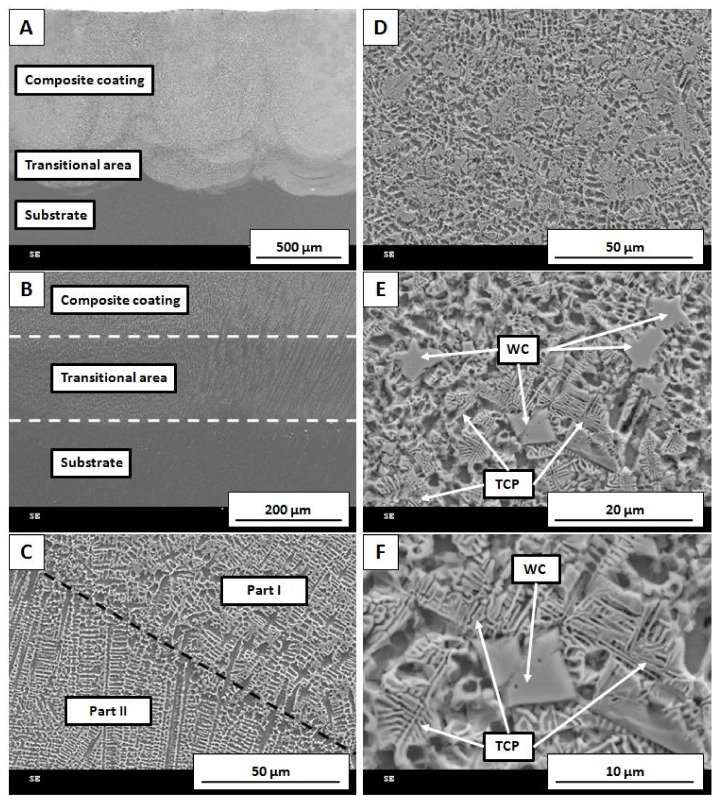
SEM images of different parts of the sample: (**A**,**B**) cross-section of the coating–substrate boundary, *(***C**) area where direction of grain growth was altered, (**D**–**F**) partially dissolved WC grains with characteristic fishbone-like structure of topologically close-packed (TCP) phases.

Figure 4 depicts the morphology of the prepared samples at various magnifications. As shown in Figure 4A,B, three areas can be recognized. The composite coating obtained by LMD has a fine uniform microstructure. The transitional area constitutes the boundary between composite coating and base material. The bottom area represents the substrate Inconel 625. Its microstructure is characterized by a larger grain size than the coating. Additionally, Figure 4B shows that the structural orientation of the coating grains tends to reflect the grain orientation in the substrate. Figure 4C presents the area where the direction of grain growth was changed because of a slightly different temperature gradient during the process.

Figure 4D–F shows the part of the sample where partially dissolved WC was visible. When compared to Figure 4C, the microstructure of the material was altered. Metallic grains were equiaxial, and WC was located at their boundaries. According to Figure 4E, it is evident that coarse WC was partially dissolved in metallic matrix, forming a typical eutectic microstructure. In Figure 4F, it can be seen that the dissolution process started at WC grain tips and propagated into the metal matrix. It formed a fishbone-like structure typical for topologically close-packed (TCP) phases.

**Figure 5:**
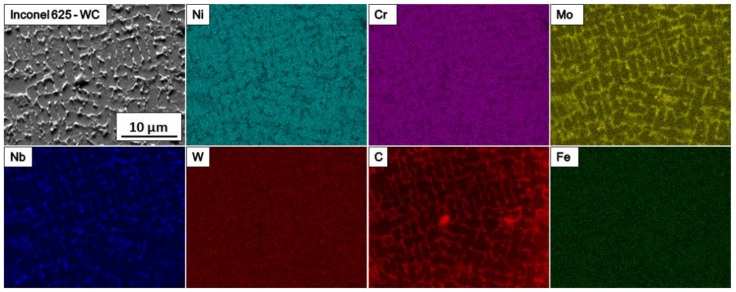
SEM-EDS elemental maps—segregation of the elements in the metal matrix composite (MMC) coating.

SEM-EDS maps are shown in Figure 5. An increased amount of Mo, Nb, and C is visible at grain boundaries. This is the result of element segregation that normally occurs after long exposure to elevated temperature. The solidification process of Inconel alloys exhibits the tendency of individual elements to segregate into a dendrite axis and interdendritic spaces depending on their partition coefficient k.

The K coefficient is experimentally determined by X-ray microanalysis of the element concentration in the dendrite core or cell (C_core_). It represents the average concentration (C_0_) of a given element in the analyzed coating area according to the following equation [9,22,26]:

k = C_core_/C_0_, (1)

The elements for which parameter k < 1 tends to segregate at interdendritic spaces, and those for which the value of parameter k > 1 diffuses into the dendrite axis. The k coefficient for Fe, Cr, W, and Co is close to 1, in comparison to 0.80 ÷ 0.85 for Mo and about 0.5 for Nb. Phases formed from elements that tend to segregate at the interdendritic spaces crystallize the last. As a result of rapid cooling, these elements often form M_x_C_y_ carbides or intermetallic phases during the eutectic reaction. Rapid cooling during the LMD process leads to grain refinement, which results in a fine microstructure.

Because of the high temperature of LMD and rapid cooling, deposited material remained in a non-equilibrium state, which allowed for the formation of TCP phases at grain boundaries. They mostly contained Mo, Nb and C (Figure 5). Typically, TCP phases are formed in nickel alloys after long heat treatment; however, the nature of laser deposition technology and the addition of ceramics in WC form induced their appearance in the coating. Main alloying elements Ni and Cr were present in the metal matrix, while Fe and W were spread equally throughout the whole volume of sample. This shows that W diffused inside the metal matrix due to high temperature occurring.

**Figure 6:**
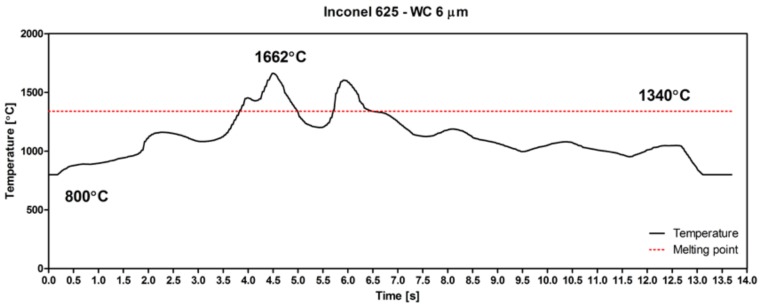
Temperature curve of deposition of a single layer of Inconel 625–WC coating during laser metal deposition.

As seen in Figure 6, the highest LMD temperature reached 1662 °C. This is much higher than the melting point of Inconel 625, 1340 °C. Thanks to the excellent wettability of WC by Ni, the appearance of a nickel-based liquid was the reason for ceramic particle dissolution. Sudden changes in temperature caused the fast melting of the Inconel 625–WC powder mixture, followed by recrystallization due to rapid solidification.

**Figure 7:**
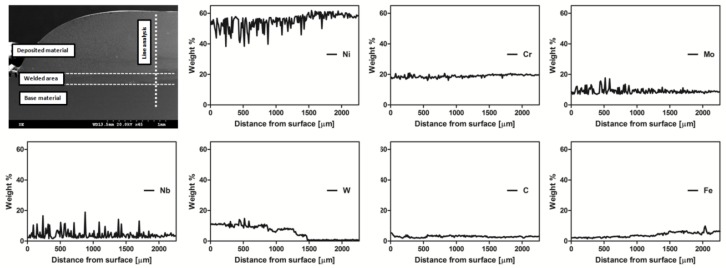
SEM images showing edge of prepared MMC sample.

Figure 7 presents the element line distribution in the material. The measured thickness of the coating was about 1200 µm. The line analysis of the cross-section shows differences in the content of the elements depending on the distance from the sample surface. The amount of Ni in the coating was 50 wt%, while in the substrate material it was 60 wt%. Chromium level remained at about 18 wt% regardless of distance from the sample surface. Mo content slightly deviated from the average, with a level in the coating of 8 wt%–9 wt%. The amount of Nb did not exhibit any significant differences depending on the transition from coating to substrate. Deviations for both Mo and Nb were caused by element segregation, which occurred mostly in the part of the sample affected by laser processing. W was present only in the coating and transitional area. It stayed at about 10 wt%, decreasing slightly with a larger distance from the sample surface, and completely disappeared at about 1500 µm. Carbon content was the same in the whole volume of the sample. The amount of Fe was very small in the coating and slightly rose in the transitional area and substrate.

**Figure 8:**
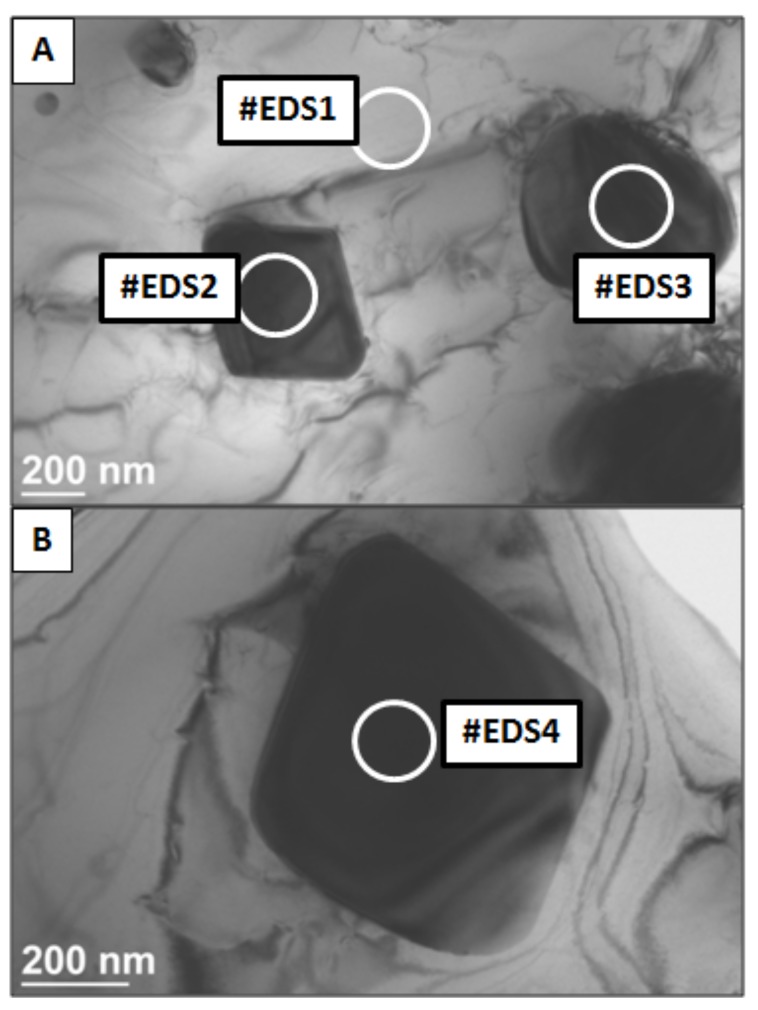
Transmission electron microscopy (TEM) image with four areas analyzed by EDS: (**A**) small precipitates (**B**) large precipitate.

**Figure 9:**
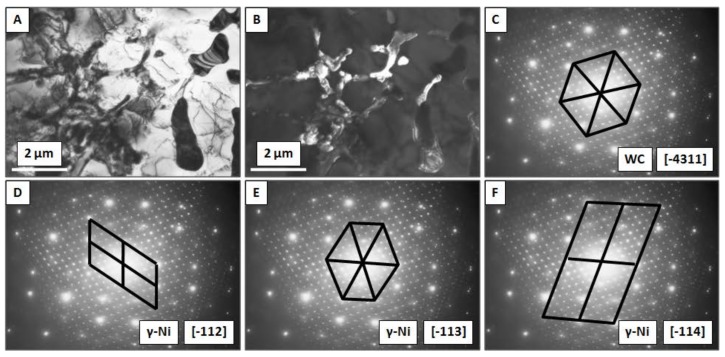
TEM images showing WC dissolution in the γ-Ni matrix: (**A**) bright-field image, (**B**) dark-field image, (**C**) diffraction pattern of WC with [-4311] crystallographic orientation, (**D**–**F**) diffraction pattern of γ-Ni grains with [-112], [-113], [-114] crystallographic orientation.

The EDS point analysis performed by TEM is shown in Figure 8 and Figure 9. Four different areas were investigated. Element concentration is presented in Table 2. High amount of Ni in area #EDS1was about two times higher in comparison to three other investigated regions. This states that light-gray areas in the images represents metal matrix of the composite. Points #EDS2, #EDS3 and #EDS4 contains high amount of W: 44, 50, and 53 wt% respectively. Together with a slightly increased concentration of Mo and Nb this indicates that dark areas represent undissolved WC particles. The presence of Nb and Mo resulted from element segregation at the grain boundaries during solidification of the material.

Figure 9 presents partially dissolved WC particles located at the grain boundaries of the metal matrix. Figure 9A depicts the bright field image of material. The light-gray area is identified as γ-Ni, while dark-gray areas represent the WC and TCP phases. It can be observed that carbides have smooth edges that were caused by partial dissolution in the Ni. The dark field of the same area is shown in the Figure 9B, with a complex diffraction pattern in Figure 9C–F. The main reflexes originated from γ-Ni matrix grains with FCC crystallographic orientation of [-112], [-113], [-114] as presented in Figure 9D–F. This indicates that matrix-grain growth proceeded similarly. The differences were caused by the introduction of additional particles into the material. Secondary reflexes visible in Figure 9C originated from WC with a crystallographic orientation of [-4311].

**Figure 10:**
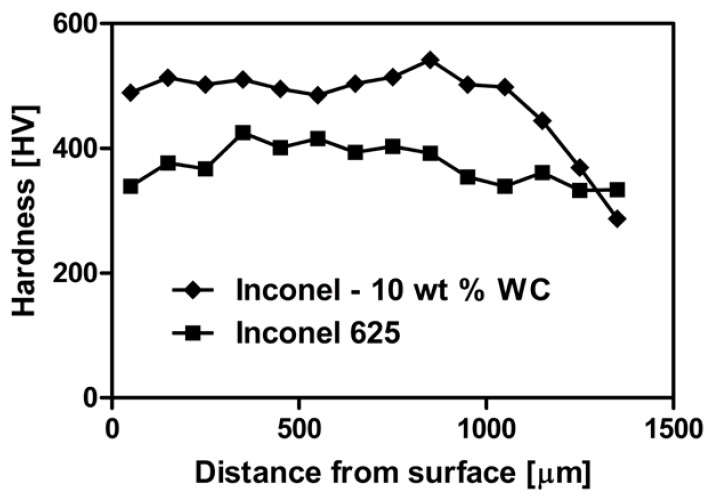
Vickers hardness of Inconel 625–WC coating and pure Inconel 625.

As shown in Figure 10, the hardness of the obtained composite coating was higher than in pure Inconel 625. The presence of carbide particles significantly increased the hardness of laser-clad material from 399 ± 14 HV_0.2_ for Inconel 625, to 502 ± 20 HV_0.2_ for the composite. Introduction of hard WC particles and the formation of a small amount of TCP phases at the grain boundaries increased the overall hardness of the composite. For Inconel 625, sample hardness measured in a distance of 0 to 400 μm from the surface was below average. In deeper parts of the coating, it rose to a maximum of 425 HV_0.2_ and gradually declined, with the distance to a minimum of 334 HV_0.2_ at about 1400 μm. For the Inconel 625–WC composite, hardness was constant, in the range of 0–800 μm distance, where it increased to a maximum of 542 HV_0.2_. It was followed by a smooth decrease to 288 HV_0.2_. This emphasizes that properties of the sample in the transitional area were affected by diffusion between two materials characterized by different hardness values. 

## 4. Discussion

The analysis of the obtained material proved that the LMD-produced Inconel 625–WC composite was crack- and pore-free. Ceramic particles were well-connected to the nickel-based matrix. TCP phases provided additional “anchoring” of the reinforcement in metal. Deposition of the Inconel 625–WC powder mixture resulted in a homogeneous distribution of ceramic particles in the obtained samples when compared to non mixed powders [27,28,29].

The results show that the introduction of WC modifies the microstructure and hardness of the obtained coating. The grain size of WC allowed for observation of interesting processes on the WC–Inconel 625 boundary. Good wettability of WC by a nickel-based alloy and rapid heating and cooling during LMD resulted in surface dissolution of the ceramics. It began in sharp tips of the grains and formed a fishbone-like eutectic structure at the metal–ceramic interface. Thanks to that, it was possible to observe how WC grains were reacting with the metal matrix. Samples were kept in a temperature above the Inconel 625 melting point for a very short time (up to 2 s). Due to rapid cooling, the sample microstructure remained partially dissolved, which is difficult to achieve when using conventional coating-deposition techniques. Because of the rapid nature of the process, the material remained in a physicochemical non-equilibriumbrium state.

The WC presence in the obtained material was revealed by TEM-EDS analysis, which confirmed the assumption that grains of selected sizes survive laser processing. TEM diffraction patterns showed that the coating microstructure is complex. Crystallographic orientation of the Inconel 625 grains is similar and differences are caused by the inclusion of other phases in the material.

The presence of the WC and TCP phases was beneficial for material’s hardness properties. The overall hardness of the coating is about 25%higher than that of pure Inconel 625 obtained by the same technique. The linear decline of hardness was observed in deeper parts of the samples because of mixing between substrate and powder mixture during laser processing. This was confirmed by SEM-EDS linear element-distribution analysis. The amount of Ni, Nb, Mo and W decreased in the transitional area in comparison to coating.

Introduction of WC caused grain-size refinement. It also strengthened the composite microstructure. However, it can negatively affect corrosion resistance [32,33]. Uniform distribution of carbide particles in the whole volume of the coating is expected to improve coating wear resistance. The appearance of TCP phases can further improve wear resistance and hardness, but weaken elastic properties when compared to nano-reinforcement [35,36,37].

## 5. Conclusions

- LMD allowed us to obtain crack- and pore-free homogeneous material.
- Initially prepared Inconel 625–WC powder mixture resulted in uniform distribution of reinforcement in the whole volume of the material.
- WC grain size of 5 μm is suitable to survive the LMD process.
- Partial dissolution of WC in nickel-based matrix resulted in the appearance of TCP phases at the ceramic–metal interface.
- Composite hardness was improved by about 25% in comparison to pure Inconel 625 obtained by the same technique and parameters.

## Figures and Tables

**Table 1:** Laser metal deposition process parameters.

Parameter	Value
Diameter of laser beam spot (µm)	500 ± 5
Wavelength of laser beam (nm)	1063 ± 10
Nominal laser power (W)	320 ± 5
Scanning speed (mm/s)	10
Powder feed rate (g/min)	7.78 ± 0.10
Number of sublayers	6
Track length (mm)	10.00 ± 0.05
Temperature of the melt pool (°C)	1662 ± 10

**Table 2:** TEM-EDS point analysis in areas shown in Figure 8.

Point	Ni	Cr	Mo	Nb	W	Fe	Total
#EDS1	73	13	3	0	10	0	100
#EDS2	35	11	8	2	44	0	100
#EDS3	29	10	8	2	50	1	100
#EDS4	30	6	7	3	53	1	100
